# COSMIN Risk of Bias tool to assess the quality of studies on reliability or measurement error of outcome measurement instruments: a Delphi study

**DOI:** 10.1186/s12874-020-01179-5

**Published:** 2020-12-03

**Authors:** L. B. Mokkink, M. Boers, C. P. M. van der Vleuten, L. M. Bouter, J. Alonso, D. L. Patrick, H. C. W. de Vet, C. B. Terwee

**Affiliations:** 1 Department of Epidemiology and Data Science, Amsterdam Public Health research institute, Amsterdam UMC, Vrije Universiteit Amsterdam, Amsterdam, The Netherlands; 2 Amsterdam Rheumatology and Immunology Center, Amsterdam UMC, Vrije Universiteit Amsterdam, Amsterdam, The Netherlands; 3 Department of Educational Development and Research, Faculty of Health, Medicine and Life Sciences, Maastricht University, School of Health Professions Education, Maastricht, The Netherlands; 4 Department of Philosophy, Faculty of Humanities, Vrije Universiteit Amsterdam, Amsterdam, The Netherlands; 5 Health Services Research Unit, IMIM-Hospital del Mar Medical Research Institute; CIBER Epidemiología y Salud Pública (CIBERESP); Pompeu Fabra University (UPF), Barcelona, Spain; 6 Department of Health Services, University of Washington, Seattle, WA USA

**Keywords:** Risk of Bias, Delphi study, Quality assessment, Reliability, Measurement error, Outcome measurement instruments, COSMIN

## Abstract

**Background:**

Scores on an outcome measurement instrument depend on the type and settings of the instrument used, how instructions are given to patients, how professionals administer and score the instrument, etc. The impact of all these sources of variation on scores can be assessed in studies on reliability and measurement error, if properly designed and analyzed. The aim of this study was to develop standards to assess the quality of studies on reliability and measurement error of clinician-reported outcome measurement instruments, performance-based outcome measurement instrument, and laboratory values.

**Methods:**

We conducted a 3-round Delphi study involving 52 panelists.

**Results:**

Consensus was reached on how a comprehensive research question can be deduced from the design of a reliability study to determine how the results of a study inform us about the quality of the outcome measurement instrument at issue. Consensus was reached on components of outcome measurement instruments, i.e. the potential sources of variation. Next, we reached consensus on standards on design requirements (*n* = 5), standards on preferred statistical methods for reliability (*n* = 3) and measurement error (*n* = 2), and their ratings on a four-point scale. There was one term for a component and one rating of one standard on which no consensus was reached, and therefore required a decision by the steering committee.

**Conclusion:**

We developed a tool that enables researchers with and without thorough knowledge on measurement properties to assess the quality of a study on reliability and measurement error of outcome measurement instruments.

**Supplementary Information:**

The online version contains supplementary material available at 10.1186/s12874-020-01179-5.

## Background

Outcome measurement instruments can be used to measure changes in relevant constructs within patients over time in research or clinical practice [1]. Scores of outcome measurement instruments can be influenced by many factors (so-called sources of variation), such as the time or occasion when the measurement was taken, the instructions that were given to patients, the type of device or the settings that were used [2, 3]. In a measurement protocol it is specified how the measurements should be standardized to minimize the influence of these sources of variation. For example, the Administration and scoring manual of the Multiple Sclerosis Functional Composite (MSFC) provides detailed instructions and specification for use [4]. The MSFC consists of four tasks, one being the Nine Hole Peg test (NHPT) assessing arm and hand function [5]. The required equipment (e.g. 9-HPT apparatus, stopwatch, 9-HPT Record Form), including preparation of the equipment (e.g. ‘the apparatus should be anchored on a solid table’), instructions for communication with the patient, a schedule for conducting the test with dominant and non-dominant hand, and instructions for discontinuing the test and scoring are described in this measurement protocol. Any deviations from the protocol could lead to different scores.

Reliability studies help to estimate the influence of different sources of variation on scores, in two ways. First, by studying which sources of variation are most distorting the measurement (i.e. by evaluating the measurement property *reliability*) [3]. Second, by studying the amount of error in scores in absolute terms due to sources of variation as mentioned above (i.e. by evaluating the measurement property *measurement error* - in case of categorical outcomes also called ‘agreement’) [6]. When it is possible to better standardize these sources of variation, the measurement can be improved – leading to smaller errors, and less patients required in studies to find intervention effects [7].

When using a patient-reported outcome measure (PROM), one of the most relevant source of variation that we should know is that due to time: patients complete a PROM at different time points, e.g. before and after treatment, and we want to be sure that change in scores reflect real changes and not random or systematic variation over time. This is studied in a test-retest reliability study [8]. Other sources of variation may be important for other types of instruments, such as clinician-reported outcome measurement instruments (ClinROMs) (including e.g. readings based on imaging modalities and ratings based on observations); performance-based outcome measurement instruments (PerFOMs); and biomarker outcomes – also called laboratory values [9, 10]. These measurement instruments are typically more complex as more sources of variation can potentially influence the scores. More sources of variation complicate the design, analysis, and reporting of studies on reliability and measurement error. Depending on which sources of variation are considered, different research questions can be investigated. For example, intra-rater reliability is assessed when it is studied whether the measurement results differ when they are assessed more than once by the same rater; inter-rater reliability is assessed when it is studied whether the measurement results differ when they are assessed by different raters; more complex designs can assess whether measurement results differ when they are assessed more than once by different raters at different time points, or with different equipment, etc. Also, different research questions can be investigated depending on what part of the measurement instrument (or measurement procedure) is repeated in the reliability study: i.e. a different research question is studied when the whole measurement procedure is repeated or when only a part of the measurement procedure (e.g. only the interpretation of images) is repeated.

High quality studies on measurement error and reliability are needed to get insight in the influence of different sources of variation on measurements and scores. To evaluate the quality of studies on reliability and measurement error is a challenging task. We previously developed the COnsensus-based Standards for the selection of health Measurement INstruments (COSMIN) Risk of Bias checklist to assess the quality of studies on measurement properties of PROMs [11] (updated in 2018 [12], see also [13]). The COSMIN Risk of Bias checklist includes standards on design requirements and preferred statistical methods organized in boxes per measurement property.

In this study, we aimed to extend the COSMIN standards to assess the quality of studies on reliability and measurement error of ClinROMs, PerFOMs and laboratory values used in health care and research. More specifically, we aimed to develop a new COSMIN Risk of Bias tool to transparently and systematically determine (1) how the results of a study on reliability or measurement error can inform us about the quality of these types of outcome measurement instruments used in health care and research, and (2) whether we can trust the result obtained in the study through an assessment of its risk of bias. The target user of the Risk of Bias tool is a clinician or researcher who may or may not be familiar with all aspects of reliability, and who needs to understand reliability studies to select outcome measurement instruments. To develop the tool, we conducted a Delphi study to reach consensus among a group of international researchers with expertise on reliability and measurement error studies. A Delphi study is a method to structure discussion and come to consensus in opinion among a group of experts, by means of a series of surveys [14]. This is especially useful when issues cannot be empirically studied. In addition, by performing an online study, experts from around the world were able to participate.

## Methods

### Design of the study

This Delphi study consisted of three online survey rounds. In each round, we asked panelists to rate their agreement with each of a set of proposals. In addition, we asked reasons for each rating, to better understand the opinions of panelist and improve proposals in the next round. Responses were analyzed anonymously, and all responses were included in a separate feedback report per round (all documents are available at https://osf.io/6fnw3). We used Survalyzer (Survalyzer AG, Utrecht, the Netherlands) to create and disseminate the surveys.

### Preparation of the Delphi study

The proposals were based on a literature search and are in line with current COSMIN terminology and the Risk of Bias checklist for PROMs [12, 15]. We searched for systematic reviews on the measurement properties of ClinROMs, PerFOMs or laboratory values in the COSMIN database of systematic reviews on outcome measurement instruments (https://database.cosmin.nl/). This database contains systematic reviews published in PubMed and EMBASE on the quality of outcome measurement instruments on any health aspect (for the search strategy, see the manual of the COSMIN database available at https://cosmin.nl). The database was up-to-date until March 2016 when we selected reviews on the specified types of outcome measurement instruments to inform and inspire us for this Delphi study. From each review we extracted any standard that was used to assess the quality of the design or the appropriateness of statistical methods used of the included studies on reliability or measurement error in the review. All these standards were ordered and merged when possible. This overview was used as input into the Delphi study. The questionnaire for each round was written by one of the authors (LB) and carefully discussed within a subgroup of the steering committee (LB, CB, HdV, and MB), in consultation with others (see acknowledgment), and checked and approved by the whole steering committee (all authors).

### Panelists

We aimed to include persons with expertise in complex studies on reliability and measurement error of outcome measurement instruments used in any medical field. We searched in PubMed and EMBASE for (co-) authors who published at least 4 studies applying generalizability theory, as complex reliability and measurement error studies often need to use generalizability (G-) coefficients (which are extended Intraclass Correlation Coefficients (ICCs)) [3, 16]. In collaboration with a clinical librarian we developed search strings for PUBMED and EMBASE using terms about Generalizability theory and source of variance to identify these authors (Additional file 1). In addition, we invited authors of methodological publications on reliability and measurement error, and representatives of scientific organizations focusing on improving outcome selection such as the International Society of Quality of Life Research (ISOQOL - specifically via the ISOQOL psychometrics Special Interest Group), the International Society for Pharmacoeconomics and Outcomes Research (ISPOR), Core Outcome Measures in Effectiveness Trials (COMET) initiative, and Outcome Measures in Rheumatology (OMERACT). We invited people from various health care fields and countries.

Based on our experience, we anticipated that at the most 50% of the invited persons would participate in at least one round. Therefore, we invited approximately 150 people to ensure saturation in arguments.

### Content of the rounds

**Fig. 1:**
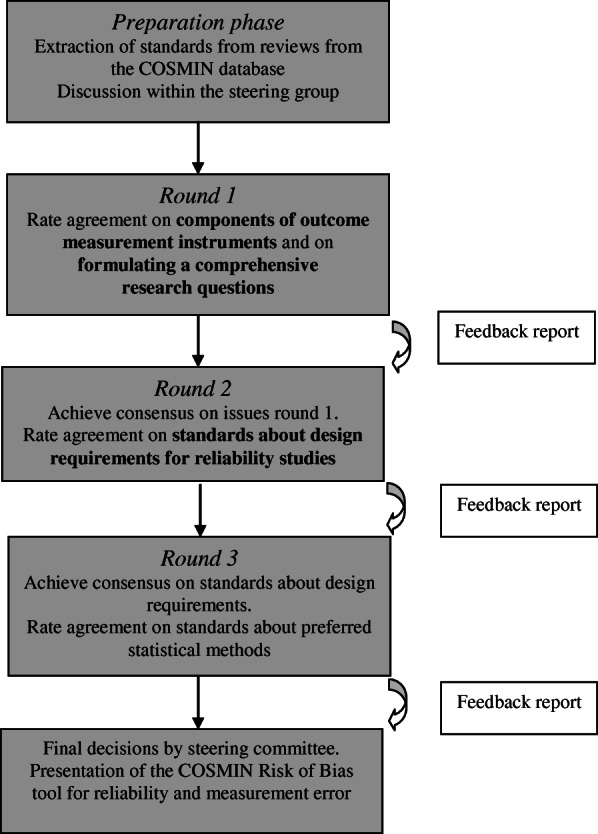
Content of the Delphi study

In round 1 (see Fig. 1), we discussed the different components of outcome measurement instruments as potential sources of variation in a reliability study. Also, we discussed elements that together make up an optimally comprehensive research question, and how to construe the research question if it is not clearly formulated in the publication.

In round 2 we aimed to reach consensus on the issues left from round 1 based on previous ratings and feedback. Based on the comments in round 1 we decided to make a separate set of components for measurement instruments of biological samples (i.e. laboratory values), and a set for other measurement instruments (i.e. ClinROMs and PerFOMs). We also proposed standards on design requirements and preferred statistical methods.

In round 3 we aimed to reach final consensus. Issues without consensus after round 3 were resolved by the steering committee.

In the Risk of Bias tool, each standard will be scored on a four-point rating system (i.e. ‘very good’, ‘adequate’, ‘doubtful’, or ‘inadequate’) in line with the COSMIN Risk of Bias checklist for PROMs [12]. In general, a standard is rated as ‘very good’ when there is evidence that the standard is met, or when a preferred method was optimally used; ‘adequate’ when it is assumable that the standard is met, or when the preferred method was used, but it was not optimally applied; ‘doubtful’ when it is unclear whether or not the standard is met or unclear if a preferred method was used; and ‘inadequate’ when there is evidence provided that the standard is not met or when the preferred method was not used. Decided a priori by the steering committee, we only discussed ratings for what constitutes very good, adequate, doubtful or inadequate preferred statistical methods. The ratings for what constitutes very good, adequate, doubtful or inadequate design requirements were not discussed, as these were adopted from the COSMIN Risk of Bias checklist for assessing studies on measurement properties of PROMs [12]. In every COSMIN box a standard about ‘other methodological flaws’ is included by default. In this tool, we also included this standard, without discussing it.

### Analyses

Agreement was rated on a five-point Likert scale (i.e. strongly disagree to strongly agree, with ‘no opinion’ in the middle). In addition, a response option ‘no expertise’ was added to each question. Consensus was reached when at least 67% of the panelists agreed or strongly agreed with a proposal (the same criterion as used in previous COSMIN Delphi studies [15, 17]) – panelist who scored ‘no expertise’ were not taken into account in the calculation for consensus for the specific item. We chose this criterion as it indicated that a substantial part of the panelists agreed to a proposal, whilst retaining room for panelists with dissenting views. If less than 67% agreement was reached on a proposal, it returned in the next round, with pro and contra arguments of panelists, and an alternative proposal. LM read all arguments and made the summary of arguments, all arguments were provided in the feedback reports and sent to all panelists and the steering committee members. Promising proposals for improvement were also posted in the next round even if consensus had been reached. When no consensus had been reached after three rounds, a steering committee – consisting of all authors or this article - made the final decision. The steering committee was also responsible for the selection of potential panelists, the content of each round and each feedback report, the analyses of responses, and the reporting of the study. The steering committee members did not act as panelist.

Based on all consensus and decisions, the steering committee developed the ‘COSMIN Risk of Bias tool to assess the quality of studies on reliability or measurement error of outcome measurement instruments’ and the ‘COSMIN Risk of Bias tool to assess the quality of a study on reliability or measurement error of outcome measurement instruments – User manual’, avaliable at https://cosmin.nl.

## Results

### Panelists

We invited 161 panelists to participate in round 1 and 2, of which 52 people (32%) (at least partly) responded to either round 1 or round 2. Forty-five (87%) invited panelists completed round 1 (and three people partially completed this round), and 41 (79%) invited panelists completed round 2 (another five partially). Round 3 was only sent to the 58 panelists who at least opened the link to any of the previous rounds; 52 of them participated in round 1 or round 2, and six panelists opened round 1 or 2, but did not respond to any proposal; 39 (75%) of the 58 invited panelists completed the third round. Thirty-six panelists (69%) completed all three rounds, 10 (19%) completed two rounds, and 6 (12%) completed one round. See Table 1 for descriptive information on the 52 panelists who participated.

**Table 1:** Descriptive information of the panelists (*n* = 52)

Country in which they mainly work	The Netherlands	14
	USA	10
	Canada	6
	UK	6
	Australia	3
	Denmark	2
	France	2
	Germany	2
	Switzerland	2
	Belgium	1
	Finland	1
	Hong Kong	1
	Italy	1
	Spain	1
Reason for invitation^a^	PubMed or EMBASE search	4
	Author of review on non-PROMs	3
	Author of methodological paper on reliability	16
	Representative of relevant organization	18
	Own network only	9
	Nominated by invited panelist	4
Professional background^a, b^	Methodologist	25
	Psychometrician	18
	Epidemiologist	17
	(Bio)statistician	15
	Allied health care professional	10
	Medical doctor	7
	(Clinical) psychologist	4
	Clinimetrician	2
	Other	3
Ever used one of the COSMIN tools?	Yes	29
	No	23

^a^ multiple answers allowed, ^b^ 56% of the panelists indicated to have more than one profession (up to 5)

### Preparatory input to the Delphi study

In the COSMIN database of systematic reviews of outcome measurement instruments 174 reviews were found that included ClinROMs, PerFOMs or laboratory values. Of these, 103 reviews described standards on any measurement property to assess the quality of the study design or the statistical methods used, of which 30 of these reviews provided standards specifically for reliability and measurement error studies (see Additional file 1). Through references in these reviews, we found three methodological papers describing a relevant checklist or guideline [18–20], one extraction form [21], and one additional systematic review [22]. All standards from this literature were used as input in our Delphi study. Important themes included in these standards were the standardization of the application of the instrument (e.g. instructions about specific equipment and settings that should be used, the environment, the professionals involved (e.g. training), etc.), independency and blinding, stability of patients, time interval, and statistical methods. We tested these items on three published studies in which generalizability theory was applied [23–25], and subsequently we realized that the research questions in these papers were not specific enough to assess whether the chosen design and statistical models were appropriate. Based on these experiences we felt the need to disentangle steps in the process of assessing the quality of a study on reliability or measurement error, into (1) understanding how exactly results of a study informed us about the quality of an instrument, and (2) assessing the quality of the study. As a basic foundation to elaborate these two steps, we decided to first identify all general components (i.e. potential sources of variation) of a measurement instrument.

### Components of outcome measurement instruments

In round 1 we started with a list of components of outcome measurement instruments that can be considered potential sources of variation that can influence the score on the measurement instrument. Based on panelists’ suggestions and comments, in round 2 the steering committee decided to propose two sets of components, one for outcome measurement instruments that involve biological sampling (i.e. blood or urine tests, tissue biopsy), and one for those that do not (i.e. ClinROMs and PerFOMs). This was proposed because the words ‘data’ and ‘score’ that we proposed to use for specific components were not considered appropriate for laboratory values. We reached consensus to use the words ‘biological sample’ and ‘value’, respectively, for outcome measurement instruments that involve biological sampling. Except for one, we reached consensus on all terms for the components and their elaboration (see Tables 2 and 3 and Additional file 1 for an elaboration). For the remaining issue, the steering committee decided to use the term ‘determination of the value of the biological sample’ over its alternative ‘actual measurement of the value of the biological sample’.

**Table 2:** Consensus of components of outcome measurement instruments that do not involve biological sampling

Component	Consensus^**a**^ on the term (%)	Elaboration	Consensus on the elaboration (%)
Equipment	43/48 (90%) (R2^b^)	All equipment used in preparation, administration, and assigning scores	43/48 (90%) (R2)
Preparatory actions	38/46 (83%) (R2)	1. ‘First time only’ general preparatory actions, such as required expertise or training for professionals to prepare, administer, store or assign the scores 2. Specific preparatory actions for each measurement, such as • preparations of equipment by professionals^c^ • preparations of the patient^d^ by the professional • Preparations undertaken by the patients	37/46 (80%) (R2)
Unprocessed data collection	30/44 (68%) (R2)	What the patient and/or professional(s) actually do to obtain the unprocessed data	33/44 (75%) (R2)
Data processing and storage	44/44 (100%) (R2)	All actions undertaken on the unprocessed data to allow the assignment of the score	37/44 (84%) (R2)
*Remove:* ‘preparation of scoring’	39/44 (89%) (R2)		
Assignment of the score	36/44 (82%) (R2)	Methods used to transform processed data into a final score^e^ on the outcome measurement instrument.	34/44 (77%) (R2)

^a^ Consensus was set at 67% of the panelists (strongly) agreed to a proposal, the denominator can be decreased because panelists considered themselves to have ‘no expertise’ on a specific proposal or dropped-out; ^b^ R2: consensus reached in Round 2; ^c^ Professionals are those who are involved in the preparation or the performance of the measurement, in the data processing, or in the assignment of the score; this may be done by one and the same person, or by different persons; ^d^ In the COSMIN methodology we use the word ‘patient.’ However, sometimes the target population is not patients, but e.g. healthy individuals, caregivers, clinicians, or body structures (e.g. joints, or lesions). In these cases, the word patient should be read as e.g. healthy volunteer, or clinician; ^e^ The score can be further used or interpreted by converting a score to another scale, metric or classification. For example, a continuous score is classified into an ordinal score (e.g. mild/moderate/severe), a score is dichotomized into below or above a normal value, patients are classified as responder to the intervention (e.g. when their change is larger than the Minimal Important Change (MIC) value)

**Table 3:** Consensus on components of outcome measurement instruments that involve biological sampling

Component	Consensus^**a**^ on the term (%)	Elaboration	Consensus on the elaboration (%)
Equipment	See above	All equipment used in the preparation, the administration, and the determination of the values of the outcome measurement instrument	See above
Preparatory actions preceding sample collection by professionals, patients, and others (if applicable)	See above	1. General preparatory actions, such as required expertise or training for professionals to prepare, administer, store and determine the value 2. Specific preparatory actions for each measurement, such as • preparations of equipment, environment, and storage by professionals^b^ • preparation of the patient^c^ by the professional • Preparatory actions undertaken by the patients	See above
Collection of biological sample	32/38 (84%) (R2^d^)	All actions undertaken to collect the biological sample, before any sample processing	33/38 (87%)^e^ (R2)
Biological sampling processing and storage	Combining^f^ 33/35 (94%) (R3^g^) Term: 29/35 (83%) (R3)	All actions undertaken to be able to preserve, transport, and store the biological sample for determination; and, if applicable, further actions undertaken on the stored sample to be able to conduct the determination of the biological sample	35/36 (97%) (R3)
Determination of the value of the sample^5^	20/35 (57%) (R3) 31/35 (89%)^i^ (R3)	Methods used for counting or quantifying the amount of the substance or entity of interest^h^	27/36 (75%) (R3)

^a^ Consensus was set at 67% of the panelists (strongly) agreed to a proposal, the denominator can be decreased because panelists considered themselves to have ‘no expertise’ on a specific proposal or dropped-out; ^b^ Professionals are those who are involved in the preparation or the performance of the measurement, in the data processing, or in the assignment of the score; this may be done by one and the same person, or by different persons; ^c^ In the COSMIN methodology we use the word ‘patient.’ However, sometimes the target population is not patients, but e.g. healthy individuals, caregivers, clinicians, or body structures (e.g. joints, or lesions). In these cases, the word patient should be read as e.g. healthy volunteer, or clinician; ^d^ R2: consensus reached R2: consensus reached in Round 2; ^e^ After round 2 we changed the formulation, but we did not rated agreement among panelists; ^f^ In round 2 we proposed two components ‘initial processing and storage’ and ‘second processing’, which we proposed to combine in Round 3; ^g^ R3: consensus reached in Round 3; ^h^ Decision by the steering committee; ^i^ Consensus reached in R3 on the term ‘value’

### Elements of a comprehensive research question

In order to understand how exactly the result of a reliability study informs us on the quality of the measurement instrument under study, in round 1 we agreed on 7 elements that can be disentangled from the described design of the study and together form a comprehensive research question (Table 4). In round 2 we proposed an alternative wording for element 4 (see Additional file 1). As a result, agreement on this element increased from 70 to 86%.

**Table 4:** Elements of a comprehensive research question of a study on reliability or measurement error

Element of the research question		Consensus^a^ (%)
1	the **name** of the outcome measurement instrument	42/45 (93%) (R1^b^)
2	the **version** of the outcome measurement instrument or way of **operationalization** of the measurement protocol^c^	42/45 (93%) (R1) (version) 33/45 (73%) (R1) (operationalization)
3	the **construct** measured by the measurement instrument	40/45 (89%) (R1)
4	a specification whether one is interested in a **reliability parameter** (i.e. a relative parameter such as an ICC, Generalizability coefficient φ, or Kappa κ) or a **parameter of measurement error** (i.e. an absolute parameter expressed in the unit of measurement e.g. SEM, LoA or SDC; or expressed as agreement or misclassification, e.g. the percentage specific agreement).	36/42 (86%) (R2^d^)
5	a specification of the **components of the measurement instrument** that will be **repeated** (especially when only part of the measurement instrument is repeated, e.g. only assignment of the score based on the same images)	38/45 (84%) (R1)
6	a specification of the **source(s) of variation** that will be **varied**^**e**^	41/45 (91%) (R1)
7	a specification of the **patient**^**f**^ **population**^**g**^ studied	42/45 (93%) (R1)

^a^ Consensus was set at 67% of the panelists (strongly) agreed to a proposal, the denominator can be decreased because panelists considered themselves to have ‘no expertise’ on a specific proposal or dropped-out; ^b^ R1: consensus reached in Round 1; ^c^ In Generalizability theory these are the *facets of stratification* (FoS), when patients are nested in a facet [16]; ^d^ R2: consensus reached in Round 2; ^e^ In Generalizability theory these are the *random or fixed facets of generalizability (FoG)*, e.g. time or occasion, the (level of expertise of) professionals, the machines, or other components of the measurement [16]; ^f^ In the COSMIN methodology we use the word patient. However, sometimes the target population doesn’t consist of patients, but e.g. healthy individuals, caregivers, clinicians, or the body structures (e.g. joints, or lesions). In these cases, the word patient should be read as e.g. healthy volunteer, or clinician; ^g^ In Generalizability theory these are the *Object of Measurement (OoM)* or *the facet of differentiation* [16]

### Standards on design requirements of studies on reliability and measurement error

To assess reliability or measurement error of an outcome measurement instrument repeated measurements in stable patients are required. The design of a study assessing any of the two measurement properties is the same, i.e. the same data can be used for estimating reliability and measurement error. Only different statistical parameters are applied to the same data to express both measurement properties.

In round 2 we reached consensus on five standards on design requirements, referring to stable patients, appropriate time interval, similar measurement conditions, and independent measurements and scoring (Table 5). Alternative wordings for the standards 4 and 5 increased consensus for these standards from 73 and 78%, respectively, to 92% in round 3.

**Table 5:** Standards for design requirements of studies on reliability or measurement error

*Design requirements*		very good	adequate	doubtful	inadequate	NA
1	Were patients stable in the time between the repeated measurements on the construct to be measured? *Relevance: 39/40 (98%) (R2*^*a*^*); wording: 33/40 (83%) (R2)*	Yes (evidence provided)	Reasons to assume standard was met	Unclear	No (evidence provided)	NA
2	Was the time interval between the repeated measurements appropriate? *Relevance: 40/41 (98%)(R2); wording: 37/41 (90%)(R2)*	Yes		Doubtful , OR time interval not stated	No	NA
3	Were the measurement condition similar for the repeated measurements – except for the condition being evaluated as a source of variation? *Relevance: 37/41 (90%)(R2); wording: 34/41 (83%)(R2)*	Yes (evidence provided)	Reasons to assume standard was met, OR change was unavoidable	Unclear	No (evidence provided)	NA
4	Did the professional(s) administer the measurement without knowledge of scores or values of other repeated measurement(s) in the same patients? *Relevance: 38/41 (93%)(R2); wording: 27/30 (90%)(R3*^*b*^*)*	Yes (evidence provided)	Reasons to assume standard was met	Unclear	No (evidence provided)	NA
5	Did the professional(s) assign the scores or determined the values without knowledge of the scores or values of other repeated measurement(s) in the same patients? *Relevance: 38/41 (93%)(R2); wording: 27/30 (90%)(R3)*	Yes (evidence provided)	Reasons to assume standard was met	Unclear	No (evidence provided)	
6	Were there any other important flaws in the design or statistical methods of the study? ^c^	No		Minor methodological flaws	Yes	

^a^ R2: consensus reached in round 2; ^b^ R3: consensus reached in round 3; ^c^ Standard 6 and the responses of the four-point rating system were not discussed in the Delphi study

### Standards on preferred statistical methods of studies on reliability

We reached consensus on three standards (Table 6) on preferred statistical methods to assess reliability of outcome measures that have continuous, ordinal and dichotomous/nominal scores, respectively, and how these standards should be rated. Preferred statistical methods are ICCs and (weighted) Kappa. Based on suggestions by the panelists, we asked in round 3 whether we should add that when the data was non-normally distributed standard 7 for continuous scores should be rated as inadequate – for which we did not reach consensus (i.e. 54%) and this proposal was therefore not included in the standard. The most important issue was the relatively low degree of consensus on the kappa statistic as a preferred statistical methods to assess reliability of ordinal scores (standard 8 for reliability): 67% agreed in round 2 that weighted kappa was the preferred statistical method to assess reliability for ordinal scores (standards 8 for reliability), and 56% agreed in round 2 that kappa was the preferred statistical methods to assess reliability for dichotomous/nominal scores (standard 9). Issues raised included the difficulty in interpreting a kappa value, and the dependence on the prevalence of a specific outcome (i.e. the heterogeneity of the sample). Panelists recommended reporting the marginals, as well as the percentage specific agreement. However, specific agreement is considered to be a parameter of measurement error (agreement), and therefore cannot be proposed as a preferred statistical method to assess reliability. In round 3 we again proposed the (weighted) kappa as the preferred statistical method to assess reliability of ordinal scores, while acknowledging that reliability is less informative than measurement error (standard 8 for reliability), and for dichotomous/nominal scores kappa was proposed calculated for each category against the other categories combined (standard 9 for reliability). The percentage consensus for standards 8 and 9 for reliability increased up to 73 and 71%, respectively.

**Table 6:** Consensus reached on standards for preferred statistical methods for reliability

*Statistical methods*		very good	adequate	doubtful	inadequate
7	For continuous scores: was an Intraclass Correlation Coefficient (ICC)^a^ calculated? *28/35 (80%)(R2*^*b*^*)*	ICC calculated; the model or formula was described, and matches the study design^c^ and the data *30/35 (86%)(R2)*	ICC calculated but model or formula was not described or does not optimally match the study design^c^ OR Pearson or Spearman correlation coefficient calculated WITH evidence provided that no systematic difference between measurements has occurred	Pearson or Spearman correlation coefficient calculated WITHOUT evidence provided that no systematic difference between measurements has occurred *25/35 (71%) (R2)* OR WITH evidence provided that systematic difference between measurements has occurred *25/34 (74%)(R2)*	
8	For ordinal scores: was a (weighted) Kappa calculated? *26/36 (72%)(R2)*	Kappa calculated; the weighting scheme was described, and matches the study design and the data *R3: 27/36 (75%)(R3*^*d*^*)*	Kappa calculated, but weighting scheme not described or does not optimally match the study design *19/36 (53%)(R3)*		
9	For dichotomous/nominal scores: was Kappa calculated for each category against the other categories combined? *23/33 (70%)(R3)*	Kappa calculated for each category against the other categories combined			

^a^ Generalizability and Decision coefficients are ICCs; ^b^ R2: consensus reached in round 2; ^c^ Based on panelists’ suggestions the steering committee decided after round 3 to use the word ‘study design’ instead of ‘reviewer constructed research question’; ^d^ R3: consensus reached in round 3

We did not reach consensus on what is considered an adequate method to assess reliability of ordinal scores (standard 8 for reliability). In round 2 60% of the panelists agreed or strongly agreed to the proposal to rate the standard as ‘adequate’ when in a study ‘the weighted kappa was calculated, but the weighting scheme was not described’. In round 3 we proposed to rate the standard as ‘adequate’ when ‘the kappa is calculated, but weighting scheme is not described or does not optimally match the reviewer constructed research question’. This proposal was in line with the proposal for the preferred statistical method to assess reliability of continuous scores. Only 54% agreed or strongly agreed to this proposal. In round 2 62% consensus was reached on the proposal to rate a study using the unweighted kappa statistic for ordinal scores as ‘doubtful’, while in round 3 only 49% (strongly) agreed to rate a study as ‘adequate’ when the unweighted kappa statistic was used. Panelists argued that the weighted kappa is mathematically the same as the ICC. After round 3, we further discussed this issue within the steering committee, and decided to keep it as suggested in round 3 (Table 6) to be in line with the standard for continuous scores.

### Standards on preferred statistical methods of studies on measurement error

We reached consensus on two standards on preferred statistical methods to assess measurement error (Table 7). For continuous scores (standard 7 for measurement error) we reached consensus that the Standard Error of Measurement (SEM), Smallest Detectable Change (SDC), Limits of Agreement (LoA) or Coefficient of Variation (CV) were the preferred statistical methods, and for ordinal/dichotomous/nominal scores (standard 8 for measurement error) the percentage specific (e.g. positive and negative) agreement was preferred (see Table 7). In round 3 we agreed on an alternative wording for the responses of the four-point rating system of the standard for continuous scores, to be in line with the proposed wording for the standard on reliability for continuous scores.

**Table 7:** Consensus reached on standards for preferred statistical methods for measurement error (agreement)

*Statistical methods*		very good	adequate	doubtful	inadequate
7	For continuous scores: was the Standard Error of Measurement (SEM), Smallest Detectable Change (SDC), Limits of Agreement (LoA) or Coefficient of Variation (CV) calculated? *Relevance: 29/38 (76%)(R2*^*a*^*); wording: 22/32 (69%)(R3*^*b*^*); add CV: 22/30 (73%)(R3)*	SEM, SDC, LoA or CV calculated; the model or formula for the SEM/SDC is described; it matches the study design^c^ and the data *32/36 (89%)(R2)*	SEM, SDC, LoA or CV calculated, but the model or formula is not described or does not optimally match the study design and evidence provided that no systematic difference has occurred *25/34 (72%)(R2)*	SEM_consistency_ SDC_consistency_ or LoA or CV calculated, without knowledge about systematic difference or with evidence provided that systematic difference has occurred	SEM calculated based on Cronbach’s alpha *22/31 (71%)(R3)* OR using SD from another population *27/34 (79%)(R2)*
8	For dichotomous/ nominal/ ordinal scores: Was the percentage specific (e.g. positive and negative) agreement calculated? *24/35 (69%)(R2)*	% specific agreement calculated	% agreement calculated		

^a^ R2: consensus reached in round 2; ^b^ R3: consensus reached in round 3; ^c^ Based on panelists’ suggestions the steering committee decided after round 3 to use the word ‘study design’ instead of ‘reviewer constructed research question’

Sometimes Cronbach’s alpha instead of the ICC is used to calculate the measurement error with the formula $SEM={\sigma}_y\sqrt{\Big(1}- ICC\Big)$, where σ_y_ represents the standard deviation (SD) of the sample [26]. The panelists agreed this method is inadequate, because it is based on one full-scale measurement where items are considered as the repeated measurements, instead of at least two full-scale measurements using the total score in the calculation of the SEM. Moreover, Cronbach’s alpha is sometimes used inadequately, because it is calculated for a scale that is not unidimensional, or based on a formative model. In such cases the Cronbach’s alpha cannot be interpreted. Some panelists argued that this method of SEM calculation was better than nothing. With the explanation that a rating of ‘inadequate’ means that the SEM resulting from such a study can still be used, but the results are less trustworthy, 72% agreed to rate ‘a SEM calculated based on Cronbach’s alpha, or using SD from another population’ as ‘inadequate’.

In round 2 we reached 53% consensus to consider the Coefficient of Variation (CV) as the preferred statistical method to assess measurement error for scales with proportion or percentage scores. Several panelists pointed out that the CV is also frequently used for continuous scores, specifically for laboratory values. Therefore, we proposed that the CV is also an appropriate statistical method for continuous scores on measurement error (add it to standard 7 for measurement error), and reached 73% consensus.

### Term for ‘research question’

One final issue remained without consensus. In general, the statistical methods should match the research question and study design. We proposed to state that the statistical methods should match the ‘retrospectively formulated research question’ (round 2) or the ‘reviewer constructed research question’ (round 3). However, some panelists considered the term ‘retrospectively’ unclear and inappropriate as it could be interpreted that the research question was defined afterwards (while we meant that it was comprehensively formulated afterwards). The term ‘reviewer constructed research question’ was also considered unclear, as it was not very clear to whom ‘reviewer’ referred to (i.e. the one who is using the Risk of Bias tool and reviews a study). The steering committee finally decided to use the term ‘study design’ instead, and to state in the standards that the statistical methods should match the ‘study design’.

## Discussion

We developed a consensus-based ‘COSMIN Risk of Bias tool to assess the quality of studies on reliability and measurement error of outcome measurement instruments’, specifically ClinROMs, PerFOMs, and laboratory values, that are used in health care and research. It comprises two parts: (1) seven elements that make up a comprehensive research question of the study, which informs us on the quality of the outcome measurement instrument under study, and (2) standards on design requirements (*n* = 5) and preferred statistical methods of studies on reliability (*n* = 3) and measurement error (*n* = 2), which can be used to assess the quality of the study. The tool allows transparent and systematic determination of the quality of a study on reliability or measurement error. It guides assessment of the risk of bias, i.e. the level of trust we can place in the results, and whether the estimated parameter in the study is not systematically over- or underestimated. More information on the tool can be found in the user manual available on the website https://cosmin.nl.

The COSMIN Risk of Bias tool to assess the quality of a study on reliability or measurement error can be used, for example, in a systematic review of outcome measurement instruments. The COSMIN methodology for conducting systematic reviews of outcome measurement instruments [27] was developed specifically for PROMs. In general, this methodology can also be used for conducting systematic reviews of other types of outcome measurement instruments, incorporating the new Risk of Bias tool for studies on reliability or measurement error. Guidelines for how to incorporate it are described in the user manual.

The new tool was developed specifically for ClinROMs, PerFOMs, and laboratory values. This tool can also be used to assess the quality of studies on reliability or measurement error of PROMs or observer-reported outcome measures (ObsROMs), for example if it is used in a systematic review in which different types outcome measurement instruments are included. However, for the latter types of instruments the tool may seem unnecessarily complex. The first step in the tool (i.e. understanding how the results inform us on the quality of the measurement instrument under study) is often obvious, as the aim of reliability studies of PROMs and ObsROMs is most often to assess test-retest reliability or measurement error of the whole measurement instrument. The second step in the tool (assessing the quality of the study using the standards) will lead to the same rating compared to using the standards of the Risk of Bias checklist for PROMs. The first three standards on design requirements in both tools are the same. The standards 4 and 5 (i.e. about administrating the measurement and assigning scores without knowledge on other repeated measurements, respectively) that are included in the new Risk of Bias tool are usually not applicable to PROMs and ObsROMs, except when the aim is to assess whether the involvement of different proxies (e.g. the mother versus the father) influenced the score. However, this issue can also be taken into account in standard 3 of the Risk of Bias checklist for PROMs on the similarity of test conditions, or in standard 6 (additional flaws) in case the COSMIN checklist for PROMs is used. The response options for standards on preferred statistical methods in the new tool are somewhat differently formulated, but will lead to the same rating.

### Comparison with existing literature

It was our aim to develop a risk of bias tool, not a study design checklist, nor a reporting guideline. Therefore, we did not include standards referring to the relevance or generalizability of the study results. For example, we did not include standards about choices regarding the inclusion of patients or professionals (e.g. well-trained), or how the measurement procedure was carried out. In other existing checklists such standards were included. For example, the first item in the Quality Appraisal of Reliability Studies (QAREL) checklist [20] is ‘Was the test evaluated in a sample of subjects who were representative of those to whom the authors intended the results to be applied?’. This refers to the generalizability of the results, but it does not refer to the quality of the study.

Other checklists have a different scope. The Guidelines for Reporting Reliability and Agreement Studies (GRASS) are reporting guidelines [19], and therefore include items referring to the relevance and the generalizability of the study. For example items about the description of the patient population and rater population are included. The QAREL checklist [20] and the Quality Assessment of Diagnostic Accuracy Studies (QUADAS-2) [18] are checklists to assess the quality of studies on accuracy of diagnostic tests, while the COSMIN Risk of Bias tool focusses on outcome measurement instruments.

### Strength & Limitations

The strength of our study was that we focused on the arguments instead of solely on the percentage consensus reached on proposals. We valued the comments and arguments provided by the panelists highly. After each rating on each proposal we asked panelists to provide their arguments. Also, we gave the opportunity to comment on the proposals or the study in general in each round. The arguments enabled us to make better proposals in the next round. For example, we improved the formulations of standard 4 on design requirements, where we proposed to ask whether ‘professionals independently administer the repeated measurement’. Based on suggestions by panelists we changed it into whether ‘professionals administered the measurements without knowledge of the scores of other repeated measurements’. Even when we reached consensus (i.e. 67% or more of the panelists agreed or strongly agreed to a proposal) we occasionally made an alternative proposal in the next round, if valid arguments were provided which led to a better proposal. In addition, we think that panelists can better participate in the discussion when summaries of pro and contra arguments are provided. All comments were thoroughly read by one of the authors (LM), and part of them were read by one or more of the other co-authors. In addition to a full feedback report, a summary of the arguments per proposal was also given in the next round, when necessary.

There were three issues on which no consensus was achieved after three rounds, which were on the terms ‘determination of the value of the sample’, and ‘reviewer constructed research question’, and on the most adequate rating for the standard on reliability for ordinal scores. We discussed these issues in the steering committee by means of a similar approach as we did in the Delphi study. LM summarized all arguments per issue, and asked the other steering committee members to rate their agreement or preference on the proposals for these three issues and provide arguments. We think that because we received a number of arguments to facilitate our choice, we were in line with the opinions of the panelists.

The response rate of potential panelists actually participating to our study was lower than of previous COSMIN studies: 45/161 (28%) participated in round 1 in this study, while in the COSMIN Delphi study to develop the taxonomy and original Risk of Bias checklist 42/91 (46%) participated in round 1 [15] and in the COSMIN Delphi study for content validity 158/340 (46%) participated in the first round [17]. The lower response rate could to be due to the fact that in round 1 we started with a survey asking questions about the components of outcome measurement instruments, and therefore, people with a methodological background might be put off to participate to this Delphi study. As we don’t know how much expertise panelists had with the different types of outcome measurement instruments, this could have influenced in the results of the consensus on the components of measurement instruments. Also, we asked people an hour of their time per round. This might have prevented people from participating to this study. However, we received around 40 responses in each round, reached consensus within the panel on most standards and harvested many useful remarks and comments. Next, six of the 52 participating panelists responded to one round only. By personal email contact, three of them indicated that they couldn’t participate to a round due to time restrictions, and one was with maternity leave after round 1.

### Future research

In this study, we focused on ‘preferred statistical methods’, referring to methods that are appropriate for evaluating reliability or measurement error of outcome measurement instruments and are commonly used in the literature. Other methods may be appropriate as well (for example bi-factor models [28] or Multi-Trait Multi-Method (MTMM) analyses [29], or newly developed methods). It was not our intention to comprehensively describe all possible statistical methods. When these methods become common practice, the standards for statistics possibly need adaptation to accommodate newer methods.

In some of the standards on preferred statistical methods it is stated that the ICC model or formula should match the study design and the data (e.g. standards 7 for reliability and for measurement error). Statistical knowledge is required to answer this question, especially in complex situations. Text books e.g. [2, 3], or methodological papers e.g. [16, 30, 31] on ICC or Generalizability theory are available. However, these are often written in the context of psychology and education, and require extensive statistical knowledge. More accessible papers would increase the understanding and facilitate the choice for the appropriate ICC model.

## Conclusion

The COSMIN Risk of Bias tool to assess the quality of a study on reliability or measurement error aims to enable clinicians or researchers who may or may not be familiar with all aspects of reliability to assess the quality of these studies in a systematic and transparent way, for example in the context of a systematic reviews on outcome measurement instruments. Furthermore, the consensus we reached on the construction of a comprehensive research question can facilitate future researchers to better report their research question in studies on reliability or measurement error.

## Supplementary Information

**Additional file 1: Appendix 1.** Search strategies for finding potential panelists. **Appendix 2.** References for systematic reviews on measurement instruments from the COSMIN database of systematic reviews of outcome measurement instruments. **Appendix 3.** Components of outcome measurement instruments that do not involve biological sampling. **Appendix 4.** Components of outcome measurement instruments that involve biological sampling. **Appendix 5.** Formulation for element 4 of a comprehensive research question as proposed in round 1 and 2.

## Data Availability

The rounds and feedback reports generated during the current study are available in the OSF.io repository, see https://osf.io/6fnw3/.

## Abbreviations

ClinROMs: Clinician-reported outcome measurement instruments
COMET: Core Outcome Measures in Effectiveness Trials
COSMIN: COnsensus-based Standards for the selection of health Measurement INstruments
CV: Coefficient of Variation
G–coefficients: Generalizability (G) –coefficients
GRASS: Guidelines for Reporting Reliability and Agreement Studies
ICC: Intraclass Correlation Coefficient
ISOQOL: International Society of Quality of Life Research
ISPOR: The International Society for Pharmacoeconomics and Outcomes Research
LoA: Limits of Agreement
MTMM: Multi-Trait Multi-Method
MSFC: Multiple Sclorosis Functional Composite (MSFC)
NHPT: Nine Hole Peg test (NHPT
OMERACT: Outcome Measures in Rheumatology
PROM: Patient-reported outcome measure
PerFOMs: Performance-based outcome measurement instrument
SDC: Smallest Detectable Change
SEM: Standard Error of Measurement
ObsROM: Observer-reported outcome measure
QAREL: Quality Appraisal of Reliability Studies
QUADAS: Quality Assessment of Diagnostic Accuracy Studies
