# Supplementary material for: COSMIN Risk of Bias tool to assess the quality of studies on reliability or measurement error of outcome measurement instruments: a Delphi study
Source: BMC Med Res Methodol. 2020 Dec 3;20:293. doi: 10.1186/s12874-020-01179-5 (PMC7712525; doi:10.1186/s12874-020-01179-5)
Supplement: Supplementary file 1 — Additional file 1: Appendix 1. Search strategies for finding potential panelists. Appendix 2. References for systematic reviews on measurement instruments from the COSMIN database of systematic reviews of outcome measurement instruments. Appendix 3. Components of outcome measurement instruments that do not involve biological sampling. Appendix 4. Components of outcome measurement instruments that involve biological sampling. Appendix 5. Formulation for element 4 of a comprehensive research question as proposed in round 1 and 2. [file 12874_2020_1179_MOESM1_ESM.docx]

**Appendix 1. Search strategies for finding potential panelists**

Pubmed search:

G-theory[tiab] OR "G theory"[tiab] OR "generalizability theory"[tiab] OR "generalisability theory"[tiab]

EMBASE search:

‘g-theory’:ti:ab OR ‘g theory’:ti,ab OR ‘generalizability theory’:ti,ab OR ‘generalisability theory’:ti,ab

**Appendix 2. References for systematic reviews on measurement instruments from the COSMIN database of systematic reviews of outcome measurement instruments.**

1. Artero EG, Espana-Romero V, Castro-Pinero J, Ortega FB, Suni J, Castillo-Garzon MJ, et al. Reliability of field-based fitness tests in youth. Int J Sports Med. 2011;32(3):159-69.

2. Boakye M, Harkema S, Ellaway PH, Skelly AC. Quantitative testing in spinal cord injury: overview of reliability and predictive validity. J NeurosurgSpine. 2012;17(1 Suppl):141-50.

3. Brons S, van Beusichem ME, Bronkhorst EM, Draaisma J, Berge SJ, Maal TJ, et al. Methods to quantify soft-tissue based facial growth and treatment outcomes in children: a systematic review. PLoS One. 2012;7(8):e41898.

4. O'Meara SM, Bland JM, Dumville JC, Cullum NA. A systematic review of the performance of instruments designed to measure the dimensions of pressure ulcers. Wound Repair Regen. 2012;20(3):263-76.

5. Bialocerkowski A, O'Shea K, Pin TW. Psychometric properties of outcome measures for children and adolescents with brachial plexus birth palsy: a systematic review. Developmental Medicine and Child Neurology. 2013;55(12):1075-88.

6. Gelinas C, Puntillo KA, Joffe AM, Barr J. A validated approach to evaluating psychometric properties of pain assessment tools for use in nonverbal critically ill adults. Semin RespirCrit Care Med. 2013;34(2):153-68.

7. Saltzherr MS, Selles RW, Bierma-Zeinstra SM, Muradin GS, Coert JH, van Neck JW, et al. Metric properties of advanced imaging methods in osteoarthritis of the hand: a systematic review. Ann Rheum Dis. 2013.

8. Barrett EM, K.; Lewis, J. Reliability and validity of non-radiographic methods of thoracic kyphosis measurement: a systematic review. ManTher. 2014;19(1):10-7.

9. Bryant MA, Lee; Brown, Julia; Jebb, Susan; Wright, Judy; Roberts, Katharine; Nixon, Jane. Systematic review to identify and appraise outcome measures used to evaluate childhood obesity treatment interventions (CoOR): evidence of purpose, application, validity, reliability and sensitivity. Health Technol Assess. 2014;18(51):1-380.

10. Cheung PPG, Laure; Mak, Anselm; March, Lyn. Reliability of joint count assessment in rheumatoid arthritis: a systematic literature review. Semin Arthritis Rheum. 2014;43(6):721-9.

11. Crosta QR, Ward TM, Walker AJ, Peters LM. A review of pain measures for hospitalized children with cognitive impairment. J Spec Pediatr Nurs. 2014;19(2):109-18.

12. Larsen CM, Juul-Kristensen B, Lund H, Sogaard K. Measurement properties of existing clinical assessment methods evaluating scapular positioning and function. A systematic review. Physiother Theory Pract. 2014;30(7):453-82.

13. McLeod SV, Sarah. A review of 30 speech assessments in 19 languages other than English. Am J Speech Lang Pathol. 2014;23(4):708-23.

14. Register-Brown KH, L.Elliot. Reliability and validity of methods for measuring the duration of untreated psychosis: a quantitative review and meta-analysis. Schizophr Res. 2014;160(1-3):20-6.

15. Robertson SJ, Burnett AF, Cochrane J. Tests examining skill outcomes in sport: a systematic review of measurement properties and feasibility. Sports Med. 2014;44(4):501-18.

16. Sellers D, Pennington L, Mandy A, Morris C. A systematic review of ordinal scales used to classify the eating and drinking abilities of individuals with cerebral palsy. Developmental Medicine and Child Neurology. 2014;56(4):313-22.

17. Ernst MY, Albert J.; Kriston, Levente; Schonfeld, Michael H.; Vettorazzi, Eik; Fiehler, Jens. Is visual evaluation of aneurysm coiling a reliable study end point? Systematic review and meta-analysis. Stroke. 2015;46(6):1574-81.

18. Kilian SA, Laila; Goosen, Anneke; Chiliza, Bonginkosi; Phahladira, Lebogang; Emsley, Robin. Instruments measuring blunted affect in schizophrenia: a systematic review. PLoS One. 2015;10(6):e0127740.

19. Prowse AP, R; Gerdhem, P; Abbott, A. Reliability and validity of inexpensive and easily administered anthropometric clinical evaluation methods of postural asymmetry measurement in adolescent idiopathic scoliosis: a systematic review. Eur Spine J. 2015.

20. Valente ARJ, Luis M.T.; Hall, Andreia; Leahy, Margaret. Event- and interval-based measurement of stuttering: a review. Int J Lang Commun Disord. 2015;50(1):14-30.

21. van Strien AMK, C. J.; Derijks, H. J.; van Marum, R. J. Rating scales to measure side effects of antipsychotic medication: A systematic review. J Psychopharmacol. 2015;29(8):857-66.

22. Visser AWB, P.; Haugen, I. K.; Schoones, J. W.; van der Heijde, D. M.; Rosendaal, F. R.; Kloppenburg, M. Instruments Measuring Pain, Physical Function, or Patient's Global Assessment in Hand Osteoarthritis: A Systematic Literature Search. J Rheumatol. 2015;42(11):2118-34.

23. Denteneer LS, G.; De Hertogh, W.; Truijen, S.; Van Daele, U. Inter- and intra-rater reliability of clinical tests associated to functional lumbar segmental instability and motor control impairment in patients with LBP: a systematic review. Arch Phys Med Rehabil. 2016.

24. Deramore Denver BF, E.; Rosenbaum, P.; Wilkes-Gillan, S.; Imms, C. Measurement of visual ability in children with cerebral palsy: a systematic review. Dev Med Child Neurol. 2016;58(10):1016-29.

25. D'Hondt N EK, H.; Pool, J. J.; Hacquebord, S. T.; Terwee, C. B.; Veeger, D. E. Reliability of Performance-Based Clinical Measurements to Assess Shoulder Girdle Kinematics and Positioning: A Systematic Review. Phys Ther. 2016.

26. Gor-Garcia-Fogeda MDCdlC, R.; Carratala Tejada, M.; Alguacil-Diego, I. M.; Molina-Rueda, F. Observational Gait Assessments in People With Neurological Disorders: A Systematic Review. Arch Phys Med Rehabil. 2016;97(1):131-40.

27. Hidding JT, Viehoff PB, Beurskens CH, van Laarhoven HW, Nijhuis-van der Sanden MW, van der Wees PJ. Measurement Properties of Instruments for Measuring of Lymphedema: Systematic Review. Phys Ther. 2016;96(12):1965-81.

28. Wesson JC, L.; Brodaty, H.; Reppermund, S. Estimating functional cognition in older adults using observational assessments of task performance in complex everyday activities: A systematic review and evaluation of measurement properties. Neurosci Biobehav Rev. 2016;68:335-60.

29. Abedi A, Mokkink LB, Zadegan SA, Paholpak P, Tamai K, Wang JC, et al. Reliability and Validity of the AOSpine Thoracolumbar Injury Classification System: A Systematic Review. Global Spine J. 2019;9(2):231-42.

30. Jaspers MEH, van Haasterecht L, van Zuijlen PPM, Mokkink LB. A systematic review on the quality of measurement techniques for the assessment of burn wound depth or healing potential. Burns. 2019;45(2):261-81.

Appendix 3. Components of outcome measurement instruments that do not involve biological sampling

| **Component** | **Elaboration** | **Examples** |
| --- | --- | --- |
| Equipment | All equipment necessary in the preparation, the administration, and the assignment of scores of the outcome measurement instrument | Questionnaire forms, computers, tablet, pen and paper; stair steps of a specific height; device or tools (such as stopwatch, probe, tube); ultrasound machine, ultrasound gels, MRI scanner; software. |
| Preparatory actions preceding raw data collection by professionals, patients, and others (if applicable) | 1. General preparatory actions, such as required expertise or training for professionals to prepare, administer, store or assign the scores  2. Specific preparatory actions  for each measurement, such as   - preparations of equipment, environment, storage by professionals^a^ - preparations of the patient^b^  by the professional - Preparations undertaken by the patients | Training, education or experience required, certification.  Preparation of equipment: calibration of device/equipment, adjust settings of the machine.  Preparation of the environment: light conditions, room temperature, humidity, specific length of a walking track.  Preparation for storage: design database and logbook  Provide general and preparatory instructions for the patients, such as explaining the tasks/action that need to be performed including time schedule, safety issues and side effects; instructions on diet (e.g. use of caffeine), clothing (e.g. comfortable shoes, no jewelry, glasses or devices), performance during tests (e.g. perform a task as usual; try to walk as fast as you can; lie as calm as possible); set some training or perform a familiarization session.  Attaching electrodes to the body, injection with radioactive substance or contrast dye, positioning the patient, applying ultrasound gel.  Listen to and understanding the instructions provided; adherence to the preparatory instructions such as fasting, resting, taking medication, bowel preparation, exercising, shaving. |
|  |  |  |
| Collection of raw data | All actions undertaken by patient and professional(s) to collect the data, before any data processing | The patient completing questions at home, or at the hospital; or performing the tasks; the rater observing or timing the performance; switching the imaging device on and off; positioning and moving the ultrasound probe. |
| Data processing and storage | All actions undertaken on the raw data to store it in a usable (electronic) form for later data manipulation (such as score assignment or statistical analysis) | The digitally converted signal of a specific body MRI scan which is temporarily stored in the K-space, is sent to an image processor where a mathematical formula (i.e. Fourier transformation) is applied, leading to an image which is displayed on a monitor and saved on a computer;  Other examples: answers of question items are recorded on e.g. paper forms and stored or Likert scale format response options are converted into a 0-4 score and directly entered in a computer database. Performance of data quality checks e.g. double entry or validation checks on the stored/entered data. |
| Assignment of the score(s) | Methods used to convert processed data into a score^c^ that constitutes the outcome measurement instrument. | A calculation of a mathematical formula or the application of a scorings algorithm (e.g. a set of rules to be followed) to the processed data; a clinician selects the specific images and judges the severity and quantity of e.g. lesions on the set of images or compares it to a reference; scores adjusted for e.g. missing data or patients using devices such as mobility aids. |

^a^ Professionals are those who are involved in the preparation or the performance of the measurement, in the data processing, or in the assignment of the score; this may be done by one and the same person, or by different persons; ^b^ In the COSMIN methodology we use the word ‘patient.’ However, sometimes the target population is not patients, but e.g. healthy individuals, caregivers, clinicians, or body structures (e.g. joints, or lesions). In these cases, the word patient should be read as e.g. healthy volunteer, or clinician; ^c^ The score can be further used or interpreted by converting a score to another scale, metric or classification. For example, a continuous score is classified into an ordinal score (e.g. mild/moderate/severe), a score is dichotomized into below or above a normal value, patients are classified as responder to the intervention (e.g. when their change is larger than the Minimal Important Change (MIC) value).

Appendix 4. Components of outcome measurement instruments that involve biological sampling

| **Component** | **Elaboration** | **Examples** |
| --- | --- | --- |
| Equipment | All equipment used in the preparation, the administration, and the determination of the values of the outcome measurement instrument | Collection tools, such as vena puncture set, biopsy tool; material containers, such as for blood plasma (EDTA of heparin tube), for tissue (container for frozen specimens for immunofluorescence, jar filled with formalin), for urine collection (sterile, screw-top container), for standard microscopic tissue evaluation (fluid or tissue for culture (sterile jar)); laboratory equipment such as centrifuges, cabinets, and chromatography systems, computers, software. |
| Preparatory actions preceding sample collection by professionals, patients, and others (if applicable) | 1. General preparatory actions, such as required expertise or training for professionals to prepare, administer, store and determine the value | Training, education or experience required, certification. |
|  | 2. Specific preparatory actions for each measurement, such as   - preparations of equipment, environment, and storage by professionals^a^ - preparation of the patient^b^ by the professional - Preparatory actions undertaken by the patients | Preparation of equipment: calibration of device/equipment, adjust settings of the machine.  Preparation of the environment: light conditions, room temperature, humidity.  Preparation of storage: set-up all equipment for storage.  Provide general and preparatory instructions to the patients, such as explaining the measurement procedure including safety issues and side effects; instructions on diet; insertion and withdrawal of a catheter into a blood vessel.  Listen to and understanding the instructions provided; adherence to the preparatory instructions such as fasting, resting, taking medication, exercising, shaving, washing of hands. |
| Collection of biological sample | All actions undertaken to collect the biological sample, before any sample processing | Taking a blood sample or tissue biopsy, collection of a sample of urine ‘mid-stream’ in a container. |
| Biological sampling processing and storage | All actions undertaken to be able to preserve, transport, and store the biological sample for determination; and, if applicable, further actions undertaken on the stored sample to be able to conduct the determination of the biological sample | Initial reaction of material to reagent in container (e.g. anticoagulation by heparin). Blood is decomposed (by gravity) into plasma and blood cells, and stored at a specific temperature.  Tissue is snap frozen by immersion in liquid nitrogen, or fixed in formalin embedded in/processed to paraffin for long-term storage.  Blood is collected in a tube containing an aqueous solution tetrasodium salt of ethylenediaminetetraacetic acid (EDTA) and mixed with air to lyse the erythrocytes and convert hemoglobin to oxyhemoglobin.  Cut sections or prepare a smear on a slide, tissues are stained by immunofluorescent markers specific for certain surface antigens.  Screw the lid of the urine container shut, put in a sealed plastic bag and store it in the fridge at around 4 degrees Celsius, for max. 24 hours. |
| Determination of the value of the biological sample | Methods used for counting or quantifying the amount of the substance or entity of interest^c^ | The absorbance of oxyhemoglobin at 540 nm through spectrophotometry quantifies the hemoglobin concentration in the sample.  The presence of the marker on the cell surface is detected and quantified by fluorescence signal intensity.  Rater observes each slide and counts positive cells in an area.  A calculation or the application of a mathematical formula to the prepared sample. |

^a^ Professionals are those who are involved in the preparation or the performance of the measurement, in the data processing, or in the assignment of the score; this may be done by one and the same person, or by different persons; ^b^ In the COSMIN methodology we use the word ‘patient.’ However, sometimes the target population is not patients, but e.g. healthy individuals, caregivers, clinicians, or body structures (e.g. joints, or lesions). In these cases, the word patient should be read as e.g. healthy volunteer, or clinician; ^c^ The value can be further processed into a clinical score, if applicable, by a linear or semi-quantitative conversion. For example, a continuous score is classified into an ordinal score (e.g. mild/moderate/severe), a scores is dichotomized into below or above a normal value, patients are classified as responder on treatment (e.g. when their change is larger than the Minimal Important Change (MIC) value). As no noise will occur from this conversion, this is not a potential source of variance, but rather an interpretation of the value. Therefore we do not include this phase in the components for outcome measurement instruments that involve biological materials.

Appendix 5. Formulation for element 4 of a comprehensive research question as proposed in round 1 and 2.

| Round 1 | a specification whether one is interested in a **ratio-parameter** (referring to reliability or generalizability; e.g. ICC, Generalizability coefficient φ, Kappa) or a **parameter expressed in the unit of measurement** (referring to the measurement error or agreement; e.g. SEM, SDC, or percentage specific agreement). |
| --- | --- |
| Round 2 | a specification whether one is interested in a **reliability parameter** (i.e. a relative parameter such as an ICC, Generalizability coefficient φ, or Kappa κ) or a **parameter of measurement error** (i.e. an absolute parameter expressed in the unit of measurement e.g. SEM, LoA or SDC; or expressed as agreement or misclassification, e.g. the percentage specific agreement). |
